# SG-APSIC1100: Healthcare-associated infections in COVID-19 patients in Vietnam: Are we able to respond better?

**DOI:** 10.1017/ash.2023.53

**Published:** 2023-03-16

**Authors:** Thu Truong Anh, Dao Xuan Co, Do Ngoc Son, Pham The Thach, Luong Quoc Chinh, Huynh Xuan Nghiem, Nguyen Dai Vinh, Truong Thai Phuong, Pham Hong Nhung, Le Duc Nhan, Tran Thi Dung, Tran Thi Nga, Nguyen Quang Tuan

**Affiliations:** Bach Mai Hospital, Hanoi, Vietnam; Bach Mai Hospital, Hanoi, Vietnam; Bach Mai Hospital, Hanoi, Vietnam; Bach Mai Hospital, Hanoi, Vietnam; Bach Mai Hospital, Hanoi, Vietnam; Hung Vuong Hospital, Hochiminh, Vietnam; Hoa Vang District Medical Center, Danang, Vietnam; Bach Mai Hospital, Hanoi, Vietnam; Bach Mai Hospital, Hanoi, Vietnam; Da Nang Hospital, Danang, Vietnam; Bach Mai Hospital, Hanoi, Vietnam; Bach Mai Hospital, Hanoi, Vietnam; Bach Mai Hospital, Hanoi, Vietnam

## Abstract

**Objectives:** Studies have revealed that a relatively high incidence of severe infection and mortality in COVID-19 patients is attributed to healthcare-associated infections (HAIs). We implemented a study in 2 field hospitals dedicated to COVID-19 treatment in Da Nang, Vietnam (July–August 2020), and Ho Chi Minh City, Vietnam (August–October 2021), to identify pathogens, risk factors, and outcomes associated with HAIs. **Methods:** We applied a prospective study tool to estimate HAI incidence among 1,454 patients. HAIs are diagnosed and ascertained using surveillance criteria established by the US Centers for Disease Control and Prevention. All patients hospitalized for COVID-19 for at least 2 days were enrolled in this assessment of HAI risks, pathogens, and outcomes. **Results:** Among 1,454 sampled patients, 391 patients had 423 HAIs (27.1%). The highest proportion occurred in ICUs, with 422 HAI patients (34.1%). Pneumonia (n = 331, 78.3%) and bloodstream infections (n = 55, 13.1%) were the most common HAIs. Multidrug-resistant (MDR) bacteria, such as *Klebsiella pneumonia* (27.9%) and *Acinetobacter baumannii* (25.3%), were the most commonly isolated organisms. Ventilators and central venous catheters were independently associated with HAIs. Regarding the mortality rates, 55% of deaths occurred in intensive care units. Patients with HAIs (70.3%) were twice as likely to die compared to patients without HAIs (38.8%). HAIs leading to septic shock caused almost triple mortality (n = 58, 90.6%) compared with non-HAI patients (n = 412, 38.8%). HAIs prolonged hospital stay: 24.7 days for patients with HAIs and 19.1 days for patients without HAIs (*P* < .001). **Conclusions:** Patients with COVID-19–related critical illnesses are at high risk of HAIs from multidrug-resistant (MDR) bacteria. HAIs prolong hospitalization, whereas HAIs with septic shock almost tripled mortality. Guidelines and procedures to prevent and control HAIs caused by MDR bacteria as well as training and monitoring on aseptic-compliant techniques during invasive clinical procedures are needed.

